# Job strain and the risk of severe asthma exacerbations: a meta‐analysis of individual‐participant data from 100 000 European men and women

**DOI:** 10.1111/all.12381

**Published:** 2014-04-12

**Authors:** K. Heikkilä, I. E. H. Madsen, S. T. Nyberg, E. I. Fransson, H. Westerlund, P. J. M. Westerholm, M. Virtanen, J. Vahtera, A. Väänänen, T. Theorell, S. B. Suominen, M. J. Shipley, P. Salo, R. Rugulies, J. Pentti, J. H. Pejtersen, T. Oksanen, M. Nordin, M. L. Nielsen, A. Kouvonen, A. Koskinen, M. Koskenvuo, A. Knutsson, J. E. Ferrie, N. Dragano, H. Burr, M. Borritz, J. B. Bjorner, L. Alfredsson, G. D. Batty, A. Singh‐Manoux, M. Kivimäki, the IPD‐Work Consortium

**Affiliations:** ^1^ Finnish Institute of Occupational HealthHelsinkiFinland; ^2^ National Research Centre for the Working EnvironmentCopenhagenDenmark; ^3^ Institute of Environmental MedicineKarolinska InstitutetStockholmSweden; ^4^ School of Health SciencesJönköping UniversityJönköpingSweden; ^5^ Stress Research InstituteStockholm UniversityStockholmSweden; ^6^ Occupational and Environmental MedicineUppsala UniversityUppsalaSweden; ^7^ Finnish Institute of Occupational HealthTurkuFinland; ^8^ Department of Public HealthUniversity of TurkuTurkuFinland; ^9^ Folkhälsan Research CenterHelsinkiFinland; ^10^ Nordic School of Public HealthGöteborgSweden; ^11^ Department of Epidemiology and Public HealthUniversity College LondonLondonUK; ^12^ Department of PsychologyUniversity of TurkuTurkuFinland; ^13^ Department of Public HealthDepartment of PsychologyUniversity of CopenhagenCopenhagenDenmark; ^14^ The Danish National Centre for Social ResearchCopenhagenDenmark; ^15^ Department of PsychologyUmeå UniversityUmeåSweden; ^16^ Department of Occupational and Environmental MedicineBispebjerg University HospitalCopenhagenDenmark; ^17^ School of SociologySocial Policy & Social WorkQueen's University BelfastBelfastUK; ^18^ Department of Public HealthUniversity of HelsinkiHelsinkiFinland; ^19^ Department of Health SciencesMid Sweden UniversitySundsvallSweden; ^20^ Social and Community MedicineUniversity of BristolBristolUK; ^21^ Institute for Medical SociologyMedical FacultyUniversity of DüsseldorfDüsseldorfGermany; ^22^ Federal Institute for Occupational Safety and Health (BAuA)BerlinGermany; ^23^ Centre for Cognitive Ageing and Cognitive EpidemiologyUniversity of EdinburghEdinburghUK; ^24^ Inserm U1018Centre for Research in Epidemiology and Population HealthVillejuifFrance

**Keywords:** asthma, exacerbation, job strain, psychosocial, work stress

## Abstract

**Background:**

Many patients and healthcare professionals believe that work‐related psychosocial stress, such as job strain, can make asthma worse, but this is not corroborated by empirical evidence. We investigated the associations between job strain and the incidence of severe asthma exacerbations in working‐age European men and women.

**Methods:**

We analysed individual‐level data, collected between 1985 and 2010, from 102 175 working‐age men and women in 11 prospective European studies. Job strain (a combination of high demands and low control at work) was self‐reported at baseline. Incident severe asthma exacerbations were ascertained from national hospitalization and death registries. Associations between job strain and asthma exacerbations were modelled using Cox regression and the study‐specific findings combined using random‐effects meta‐analyses.

**Results:**

During a median follow‐up of 10 years, 1 109 individuals experienced a severe asthma exacerbation (430 with asthma as the primary diagnostic code). In the age‐ and sex‐adjusted analyses, job strain was associated with an increased risk of severe asthma exacerbations defined using the primary diagnostic code (hazard ratio, HR: 1.27, 95% confidence interval, CI: 1.00, 1.61). This association attenuated towards the null after adjustment for potential confounders (HR: 1.22, 95% CI: 0.96, 1.55). No association was observed in the analyses with asthma defined using any diagnostic code (HR: 1.01, 95% CI: 0.86, 1.19)**.**

**Conclusions:**

Our findings suggest that job strain is probably not an important risk factor for severe asthma exacerbations leading to hospitalization or death.

Asthma, an intermittent inflammation of bronchioles and smooth muscle in the lungs, is among the most common chronic respiratory diseases 1. Its prevalence in the working population varies by type of employment from 1.7% to 17% 3. Although rarely lethal (the estimated age‐standardized death rate worldwide was 5.2 per 100 000 individuals in 2010), asthma is a major cause of disease burden, with the estimated years lived with disability amounting to 201 per 100 000 4. Most patients manage their disease at home, with the help of their primary care physician. However, severe asthma exacerbations do happen, leading to hospitalizations and incurring costs to the individual and the healthcare system 1. In Western Europe, the age‐ and sex‐standardized hospital admission rates for asthma in adults vary (by country and demographics) from 19 to 76 per 100 000 7.

Many triggers for asthma exacerbations, such as respiratory infections and allergens (e.g. dust, pollen and moulds) or airborne irritants (e.g. tobacco smoke, chemical fumes and pollution), are well known 1. The role of psychosocial factors in asthma exacerbations, however, is less well understood 1. One psychosocial factor that may be implicated in asthma exacerbations is stress. Stress can make individuals prone to biological and behavioural triggers such as respiratory infections 11 and smoking 12, but the evidence for a direct link between stress and asthma exacerbations is unclear 1. Work is a common source of stress in adults, and epidemiological research has shown that stress is associated with an increased risk of certain diseases 13. However, a recent review identified no studies of work‐related psychosocial stress and asthma exacerbations 8. To address this gap in the knowledge, we investigated the associations between job strain, an operationalization of work‐related psychosocial stress, and severe asthma exacerbations, defined as hospitalizations or deaths, in 100 000 European men and women.

## Methods

### Studies

We used individual‐level data from 11 independent prospective cohort studies from Finland, Sweden, Denmark and the United Kingdom. All studies are part of the ‘Individual‐participant‐data Meta‐analysis of Working Populations’ (IPD‐Work) Consortium. Studies included in the present analyses were Copenhagen Psychosocial Questionnaire I and II (COPSOQ‐I and COPSOQ‐II), Danish Work Environment Cohort Study (DWECS), Finnish Public Sector study (FPS), Health and Social Support (HeSSup), Intervention Project on Absence and Well‐being (IPAW), Burnout, Motivation and Job Satisfaction study (Danish acronym PUMA), Still Working, Whitehall II and Work Lipids and Fibrinogen (WOLF) Norrland and WOLF Stockholm. Details of the IPD‐Work Consortium and these studies have been published previously and are described in Appendix S1.

### Participants

Our analyses were based on participants who worked at study baseline and had complete data on job strain, age, sex, socioeconomic position, body mass index (BMI), smoking, alcohol intake and asthma, and no history of asthma hospitalization before the study baseline or during the first 30 days of follow‐up (Table 1). We excluded individuals with missing data on job strain or covariates (in the analyses with primary asthma diagnosis as the outcome: *n* = 8 752, 7.9%, and in the analyses with any asthma diagnosis as the outcome: *n* = 9 214, 8.3%).

**Table 1:** Study characteristics

Study, country	Baseline year	Eligible participants^a^	N with complete data^b^		N (%) job strain at baseline		N severe asthma exacerbations (N hospitalizations/N deaths)	
			Asthma as primary diagnostic code	Asthma as any diagnostic code	Asthma as primary diagnostic code	Asthma as any diagnostic code	Asthma as primary diagnostic code	Asthma as any diagnostic code
Copenhagen Psychosocial Questionnaire I (COPSOQ‐I), Denmark	1997	1853	1716	1707	348 (20.3)	348 (20.5)	22 (22/0)	22 (22/0)
Copenhagen Psychosocial Questionnaire II (COPSOQ‐II), Denmark	2004‐2005	3818	3293	3280	463 (14.1)	461 (14.1)	15 (15/0)	12 (12/0)
Danish Work Environment Cohort Study (DWECS), Denmark	2000	5606	5428	5375	1196 (22.0)	1 194 (22.2)	38 (38/0)	36 (36/0)
Finnish Public Sector (FPS), Finland	2000	48 592	44 007	43 652	7052 (16.0)	6993 (16.0)	132 (132/0)	371 (369/2)
Health and Social Support (HeSSup), Finland	1998	17 102	15 004	15 004	2644 (17.6)	2644 (17.6)	46 (46/0)	47 (46/1)
Intervention Project on Absence and Well‐being (IPAW), Denmark	1996–1997	2068	1912	1905	330 (17.3)	330 (17.3)	38 (38/0)	37 (37/0)
Burnout, Motivation and Job Satisfaction study (Danish acronym PUMA), Denmark	1999–2000	1914	1723	1716	257 (14.9)	256 (14.9)	22 (22/0)	20 (20/0)
Still Working, Finland	1986	9282	8916	8911	1390 (15.6)	1387 (15.6)	58 (58/0)	117 (117/0)
Whitehall II, United Kingdom	1985–1988	10 308	10 175	10 175	1421 (14.0)	1421 (14.0)	42 (35/4)	382 (378/4)
Work Lipids and Fibrinogen (WOLF) Norrland, Sweden	1996–1998	4718	4561	4556	581 (12.7)	579 (12.7)	8 (8/0)	21 (20/1)
Work Lipids and Fibrinogen (WOLF) Stockholm, Sweden	1992–1995	5698	5472	5464	873 (16.0)	871 (15.9)	9 (9/0)	44 (44/0)
All	1985–2005	110 959	102 207	101 745	16 555 (16.2)	16 484 (15.7)	430 (430/0)	1109 (1 101/8)

a Eligible participants were men and women who responded to the baseline questionnaire and were employed at study baseline.

b Participants with complete data on job strain, asthma and covariates.

### Job strain exposures

Job strain was ascertained using questions from the validated Job Content Questionnaire and Demand‐Control Questionnaire 15. A detailed description of the job strain measure has been published previously 17. Briefly, participants answered questions on the psychosocial aspects of their job at study baseline. For each participant, mean response scores were calculated for job demand items and job control items. High demands were defined as a score higher than the study‐specific median score; low control was defined as a score lower than the study‐specific median score. Job strain was categorized as high strain (high demands and low control), active job (high demands and high control), passive job (low demands and low control) and low‐strain job (low demands and high control). Binary job strain was defined as job strain (high demands and low control) versus no strain (all other categories combined).

### Asthma exacerbations

Severe asthma exacerbations were ascertained from national hospitalization and death registries in all studies. Asthma was defined as the International Classification of Diseases (ICD) version 9 code 493 or version 10 codes J45 or J46. 18 We investigated asthma as the primary diagnostic code and asthma as any diagnostic code. The date of the asthma exacerbation was defined as the date of hospital admission or the date of death to which asthma contributed. Hospital records were available since the 1960s in WOLF studies, 1970s in FPS, Still Working and the Danish studies, and 1997 in HeSSup.

### Potential confounders

We adjusted our analyses for age, sex, socioeconomic position, BMI, tobacco smoking and alcohol intake. Details of the ascertainment, harmonization and modelling of these are provided in Appendix S2. Briefly, information on sex and age was obtained from population registries or interview. Socioeconomic position was based on occupation ascertained from the employers' or other registers or participant‐completed questionnaires. Smoking and alcohol intake were participant‐reported. BMI (weight in kilograms divided by height in metres squared) was calculated from height and weight, which were participant‐reported or measured at baseline examination.

### Statistical analyses

We modelled job strain as binary (strain vs. no strain) and categorical (high strain, active job and passive job vs. low strain). Asthma exacerbations (hospitalizations or deaths, with asthma as the primary diagnostic code or as any diagnostic code) were modelled as binary outcomes. We modelled the associations between job strain and asthma exacerbations in each study using Cox regression, with the participant's age as the timescale. Each participant was followed from the date of their baseline assessment to the first asthma hospitalization, death or the end of the registry follow‐up, whichever occurred first. We ran age‐ and sex‐adjusted models and multivariable‐adjusted models, which were further adjusted for socioeconomic position, BMI, smoking and alcohol intake. We checked the proportional hazards assumption using the Schoenfeld test. The study‐specific effect estimates were pooled using fixed‐effect and random‐effects meta‐analyses, and heterogeneity was quantified using the I^2^ statistic 21. Both fixed‐effect and random‐effects meta‐analyses are shown in the figures for comparison, but results from random‐effects meta‐analyses are reported in the text. Meta‐regression was used to examine the impact of study‐level characteristics. Data on prebaseline hospitalizations were unavailable in Whitehall II, but we conducted sensitivity analyses to explore the impact of this (Appendix S3). All statistical analyses were conducted using Stata 11 (Stata Corporation Ltd., College Station, TX, USA) apart from study‐specific analyses in the Danish studies, which were conducted using SAS 9.2 (SAS Institute Inc., Cary, NC, USA).

## Results

The characteristics of the studies and participants are shown in Table 1 and Appendix S3, Table S1. Of the 102 207 participants, 56% were women. The proportion of participants who reported job strain at baseline varied by study from 13% to 22%. During a median follow‐up of 10 years (range: 1–24 years), 430 individuals had a severe asthma exacerbation as a primary diagnostic code and 1109 individuals had a severe asthma exacerbation as any diagnostic code (Table 1).

**Figure 1:**
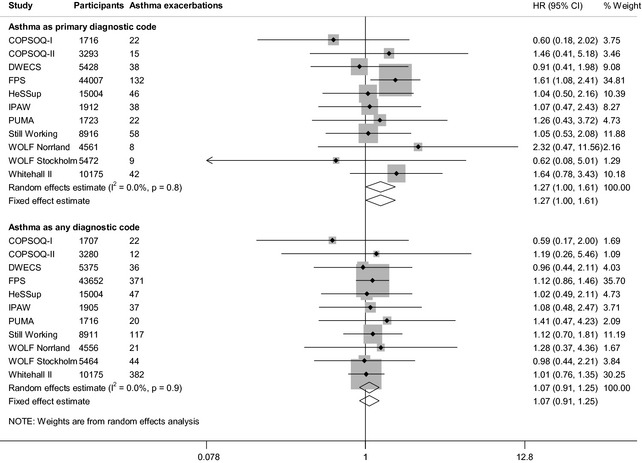
Age‐ and sex‐adjusted association between job strain and severe asthma exacerbations.

**Figure 2:**
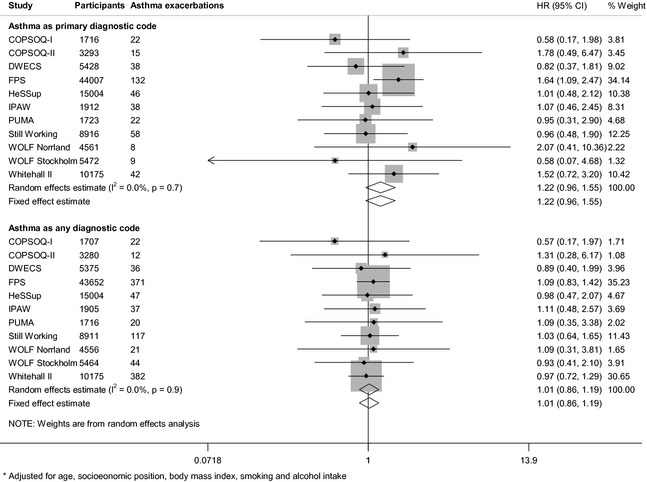
Multivariable‐adjusted *association between job strain and severe asthma exacerbations.

Job strain at baseline was associated with an increased risk of severe asthma exacerbations in the age‐ and sex‐adjusted analyses with the outcome definition based on the primary diagnostic code (HR: 1.27, 95% CI: 1.00, 1.61) (Fig. 1) However, with additional adjustment for socioeconomic position, BMI, smoking and alcohol intake, this association attenuated towards the null (HR: 1.22, 95% CI: 0.96, 1.55) (Fig. 2). Job strain was not associated with severe asthma exacerbations defined as any diagnostic code in the age‐ and sex‐adjusted analyses (HR: 1.07, 95% CI: 0.91, 1.25) or multivariable‐adjusted analyses (HR: 1.01, 95% CI: 0.86, 1.19) (Figs 1 and 2). There was little heterogeneity among the study‐specific estimates.

**Figure 3:**
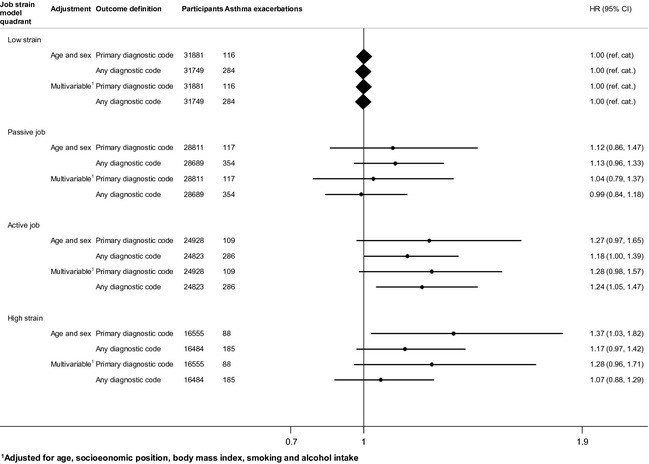
Associations between job strain model quadrants and severe asthma exacerbations.

We found no clear evidence for an association of passive job or high strain with severe asthma exacerbations (Fig. 3). The findings were similar in the analyses with asthma exacerbations defined based on primary or any diagnostic code. There was some indication of active job (high demands and high control over work) being associated with a slightly increased risk of a severe asthma exacerbation (multivariable‐adjusted HRs: asthma as primary diagnostic code: 1.28, 95% CI: 0.98, 1.57; asthma as any diagnostic code: 1.24, 95% CI: 1.05, 1.47) (Fig. 3). The study‐specific results, on which these summary estimates are based, are shown in Appendix S3 (Figs. S1–S4).

As smoking is a strong confounder to any association between work‐related stress and asthma exacerbations, we also fitted all the models stratified by baseline smoking. We found no clear evidence of job strain being associated with severe asthma exacerbations in baseline smokers or baseline nonsmokers (Appendix S3, Table S2). Results from these and other sensitivity analyses (described in Appendix S3) suggest that our findings are robust.

## Discussion

### Main findings

In our meta‐analyses of individual‐level data from over 100 000 participants from four European countries, we found no clear evidence for an association between job strain (a combination of high demands and low control over work) and the risk of severe asthma exacerbations, regardless of whether asthma was ascertained from the primary diagnostic code (HR: 1.22, 95% CI: 0.96, 1.55) or any diagnostic code (HR: 1.01, 95% CI: 0.86, 1.19). These findings suggest that job strain is probably not an important risk factor of severe asthma exacerbations leading to hospitalization or death. There was some indication that active job was associated with an up to 1.28‐fold increase in the risk of severe asthma exacerbations. These estimates were robust to adjustment for age, sex, socioeconomic position, smoking, alcohol intake and BMI, which suggests that they are not explained by unhealthy lifestyle among individuals reporting job strain.

### Our findings in context

Work‐related psychosocial stress has been reported as being associated with an increased risk of depression 14 as well as some, though not all, somatic diseases 13. Results of animal and *in vitro* studies suggest that the physiological stress response, characterized by inflammation and other changes in the immune system functions, may have an aetiological role in asthma 24. There is some evidence that stressful life events increase the risk of asthma exacerbations in children 25 and adults 26. Stress is also associated with asthma triggers, such as respiratory infections 11 and smoking 12. However, we found no association between job strain and severe asthma exacerbations, which could suggest that at population level, job strain specifically or without other life stressors does not make asthma worse.

In our analyses, having an active job (high demands combined with high control over work) was associated with a slight increase in the risk of severe asthma exacerbations. This association was not hypothesized. However, similar findings in relation to other diseases have been reported. An active job was associated with a 38% increase in the risk of myocardial infarction or stroke (95% CI: 1.07, 1.77) in the United States Women's Health Survey (*n* = 22 086) 28. Among 48 361 women in the Finnish Public Sector study, those with active jobs were more likely to have cerebrovascular disease than those with low‐strain jobs (odds ratio: 2.32, 95% CI: 1.30, 4.10) 29. Also, high decision authority, a feature of active jobs, was associated with an increase in mortality among Finnish industrial employees 30. Despite the similar associations between active job and other outcomes, we believe that the association between an active job and an increased risk of a severe asthma exacerbation requires cautious interpretation. This finding may relate to job strain (high demands and low control) and active job (high demands and high control) measuring different aspects of work‐related stress. In the original job strain theory, job strain is proposed to be detrimental to health, whereas high control over work is proposed to buffer against the adverse health effects of high demands. However, it may be that job strain captures the stressful aspects of routine work and that active job captures stress resulting from decision authority, control and responsibility, not only over one's own but also over others' work, which are more typical to managerial‐level jobs.

### Strengths and limitations

Our analyses were based on a large set of data from European men and women from a range of work settings and socioeconomic backgrounds, making the findings widely generalizable to working people in Northern and Western Europe. We investigated a validated measure of work‐related psychosocial stress, job strain, which was harmonized across the studies, thus making it reasonable to combine the study‐specific results in meta‐analyses 15. Also, using an exposure that was defined and harmonized before the acquisition of outcome data, we avoided bias arising from *post hoc* modifications of the exposure measure 17.

Our register‐based outcomes are strength as well as a limitation. Asthma exacerbations ascertained from hospitalization and death registers were based on clinical diagnoses. The specificity of the asthma diagnoses in the Danish hospitalization register is 98% 31. The specificity and positive predictive values for the majority of diseases are also reasonable in the Finnish (95%) and Swedish (85‐95%) hospitalization registers 32. Prospectively collected, register‐based outcome data cannot have been influenced by recall bias from the participants or the diagnosing physicians. However, as our analyses focused on severe asthma exacerbations, the findings cannot be extrapolated to associations between job strain and mild asthma exacerbations, which the patients can manage at home or in primary care. Also, the number of incident asthma exacerbations in the analyses using primary diagnostic codes to define asthma outcomes was smaller (n = 430) than in the analyses using any diagnostic code for asthma in the outcome definition (n = 1 109), which limited the statistical power in the first set of analyses.

Similarly, our study design has strengths as well as limitations. On one hand, our individual‐participant meta‐analyses of unpublished data were not prone to publication, reporting or citation biases, but on the other hand, our data were obtained from a collaborative research project and thus not based on a search of all potentially existing unpublished data. Another limitation is that we had no harmonized data on changes in job strain, occupation or working environments, exposure to air pollution, or the asthmatics' control of their disease and were thus unable to investigate whether these influenced our findings. Finally, although we adjusted our analyses for a number of potential confounders, it is possible that residual confounding from other unknown or unmeasured confounders has influenced our estimates or that they occurred due to chance.

### Clinical relevance

Our findings would be useful to clinicians, particularly in occupational health care, who need evidence‐based information on work‐related factors that impact on the management of asthma in adults. This information would also benefit patients, who may be concerned about the impact of stressful work on asthma. The association between active jobs and an increased risk of severe asthma exacerbations, however, would merit further research, and it would be premature to propose changes to clinical practice based on this observation. Also, further studies would help to ascertain whether other aspects of work‐related stress, such as low remuneration for demanding work or long working hours, might have an impact on asthma exacerbations.

## Conclusions

Our findings suggest that job strain is unlikely to be an important risk factor for severe asthma exacerbations. The observed association between active job and an increased risk of severe asthma exacerbations should, at this stage, be treated as a hypothesis‐generating finding.

## Author contributions

All authors participated in designing the investigation, generating hypotheses, interpreting the data and writing and critically reviewing the paper. Some authors participated in collecting the data. Katriina Heikkila analysed the data from FPS, HeSSup, Still Working, Whitehall II and WOLF Norrland and WOLF Stockholm. Ida E.H. Madsen analysed data from COPSOQ‐I, DWECS, IPAW and PUMA. Katriina Heikkila wrote the first draft of the paper with help from Mika Kivimäki.

## Ethical approval

Each constituent study in the IPD‐Work Consortium was approved by the relevant local or national ethics committees, and all participants gave informed consent to take part. Details of the ethical approval are provided in Appendix S1.

## Data access

Katriina Heikkila and Mika Kivimäki had full access to anonymized data from FPS, HeSSup, Still Working, Whitehall II and WOLF Norrland and WOLF Stockholm. Ida E.H. Madsen had full access to anonymized data from COPSOQ‐I, COPSOQ‐II, DWECS, IPAW and PUMA.

## Funding

The IPD‐Work Consortium is supported by the EU New OSH ERA research programme (funded by the Finnish Work Environment Fund, Finland), the Swedish Research Council for Working Life and Social Research, Sweden, the Danish National Research Centre for the Working Environment, Denmark, the Academy of Finland (Grant #132944), the BUPA Foundation (grant #22094477) and the Economic and Social Research Council, UK. Mika Kivimäki is supported by the National Heart, Lung and Blood Institute, NIH, US, the Medical Research Council, UK (K013351), and a professorial fellowship from the Economic and Social Research Council, UK. The funding bodies had no role in the study design, data collection and analysis, decision to publish or preparation of the manuscript.

## Conflicts of interest

Töres Theorell receives royalties for books written on various topics, including psychosocial factors, music and health, and Sweden's working life in the 1990s. Hugo Westerlund's institution has received a research grant from Saint‐Gobain Ecophon AB, a manufacturer of sound‐absorbing materials, to study the effect of such materials on stress, job satisfaction and productivity in open‐plan offices. Other authors declare no conflicts of interest.

## Supplementary Material

**Appendix S1.** Studies and participants.

**Appendix S2.** Potential confounders.

**Appendix S3.** Additional results.
