# Supplementary material for: Job strain and the risk of severe asthma exacerbations: a meta‐analysis of individual‐participant data from 100 000 European men and women
Source: Allergy. 2014 Apr 12;69(6):775–83. doi: 10.1111/all.12381 (PMC4114530; doi:10.1111/all.12381)
Supplement: Supplementary file 1 — Appendix S1. Studies and participants. [file all-69-775-s4.doc]

**Appendix 1. Studies and participants**

**Individual-participant Data Meta-analysis of Working populations (IPD-Work) Consortium**

IPD-Work Consortium is a collaborative meta-analysis project that was established at the Four Centers Initiative Meeting (a meeting of stress researchers from University College London, the Institut National de la Santé et de la Recherche in Paris, the University of Dusseldorf in Germany, and Karolinska Institutet in Stockholm) in London in 2008. IPD-Work Consortium consists of 19 prospective European cohort studies. The overarching aim of the consortium is to investigate the effect of work-related stress on chronic diseases using individual-participant data from prospective studies with a measure of work-related stress at baseline and register-based information on incident chronic diseases during follow-up.

Of the 19 studies, 11 studies were included in our meta-analyses and are described below. Six studies were excluded from the current analyses because no register data on severe asthma exacerbations were available (Belstress, GAZEL, Heinz-Nixdorf Recall study, Cooperative Health Research in the Region Augsburg (KORA) study, German Socioeconomic Panel Study (German acronym SOEP) and Netherlands Working Conditions Survey (NWCS)). Further two studies were excluded because the numbers of severe asthma exacerbations were too small to analyse (Swedish Longitudinal Occupational Survey of Health (SLOSH) and Permanent Onderzoek Leefsituatie (POLS)). It is unlikely that these reasons for exclusion would be associated with the study results or introduce bias to our meta-analyses. All studies provided individual-level data or conducted study-specific analyses according to our instructions and provided us with aggregate results. No study team refused to provide data or aggregate results.

IPD-Work Consortium used a pre-defined two-stage data acquisition protocol: in the first stage, baseline data on job strain and other indicators of work-related stress, socio-demographic factors and lifestyle-related factors were acquired, validated and harmonised (1-7). In the second stage, these data were linked to data on disease outcomes from national hospitalisation and mortality registers (8-10). Record linkage was done using personal identification numbers in the Danish, Finnish and Swedish studies (Copenhagen Psychosocial Questionnaire I and II, Danish Work Environment Cohort Study, Finnish Public Sector study, Health and Social Support , Intervention Project on Absence and Well-being , Burnout, Motivation and Job Satisfaction study, Still Working and Work Lipids and Fibrinogen Norrland and Stockholm studies). In Whitehall II, based in the United Kingdom, the linkage was done using the participants’ personal National Health Service numbers. All participants provided informed consent to record linkage.

**Studies included in the present analyses**

*Copenhagen Psychosocial Questionnaire version I (COPSOQ-I)*

COPSOQ-I is a prospective cohort study of a random sample of Danish residents selected from the Danish population register. The participants were aged 20-60 years of age and were in paid employment at the study baseline in 1997. A baseline questionnaire, information about the study and its aims and an invitation to take part was posted to 4 000 people and 2 454 individuals agreed to participate, of whom 1 853 were gainfully employed (11). In Denmark, questionnaire- and register-based studies do not require approval from the Danish National Committee on Biomedical Research Ethics (Den Centrale Videnskabetiske komité). COPSOQ-I was approved by and registered with the Danish Data protection agency (registration number: 2008 - 54 - 0553). Responding to the baseline questionnaire was taken to imply informed consent to take part.

*Copenhagen Psychosocial Questionnaire version II (COPSOQ-II)*

COPSOQ-II was carried out in 2004-2005. It included a follow up of respondents from COPSOQ I and also a representative sample of Danish residents aged 20-60 at study baseline. The questionnaire, with information about the study, was sent to 8 000 individuals from the random sample. The questionnaire could be completed using the posted questionnaire or via the internet (12). Of the 4 732 individuals who responded to the baseline questionnaire, 3 817 were gainfully employed and of these, 3 427 had data on job strain and were eligible for our meta-analyses. In Denmark, questionnaire- and register-based studies do not require ethics committee approval. COPSOQ-II was approved by and registered with the Danish Data protection agency (registration number: 2004-54-1493). Responding to the baseline questionnaire was taken to imply informed consent to take part.

*Danish Work Environment Cohort Study (DWECS)*

DWECS is a split panel survey of working age Danish people. The cohort was established in 1990, when a simple random sample of men and women, aged 18-59, was drawn from the Danish population register. The participants have been followed up at five year intervals and data from the year 2000 was used for the IPD-Work. That year 11 437 individuals were invited to participate and 8 583 agreed to do so (13, 14). Of these, 5 606 individuals were gainfully employed. In Denmark, questionnaire- and register-based studies do not require ethics committee approval. DWECS was approved by and registered with the Danish Data protection agency (registration number: 2007-54-0059). Participants were provided information about the study with the baseline questionnaire and responding was taken to imply informed consent to take part.

*Finnish Public Sector study (FPS)*

The Finnish Public Sector study is a prospective cohort study comprising the entire public sector personnel of 10 towns (municipalities) and 21 hospitals in the same geographical areas. Participants, recruited from employers' records in 2000-2002, were individuals who were employed in the study organisations at the time of the questionnaire survey (15). 48 592 individuals (9 337 men and 39 255 women, aged 17 to 65) responded to the questionnaire. Ethical approval was obtained from the ethics committees of the Finnish Institute of Occupational Health and Helsinki and Uusimaa Hospital District. According to the Finnish law, written consent is not required for survey and register-based research, as long as that participation is voluntary, and the participants have been informed about the aims of the study and possible register linkages (16). Thus, responding to the questionnaire voluntarily (having had access to information on the study aims and possible register linkages) was taken to imply written consent.

*Health and Social Support (HeSSup)*

The Health and Social Support (HeSSup) study is a prospective cohort study of a stratified random sample of the Finnish population in the following four age groups: 20–24, 30–34, 40–44, and 50–54. The participants were identified from the Finnish population register and posted an invitation to participate, along with a baseline questionnaire, in 1998 (17). Of the 25 898 respondents 17 102 were gainfully employed. Turku University Central Hospital Ethics Committee approved the study. All participants gave written informed consent to take part.

*Intervention Project on Absence and Well-being (IPAW)*

IPAW is a 5-year psychosocial work environment intervention study including 22 intervention and 30 control work places in three organisations (a large pharmaceutical company, municipal technical services and municipal nursing homes) in Copenhagen, Denmark (18, 19). The baseline questionnaire was posted to all the employees at the selected work-sites between 1996 and 1997. Of the 2 721 employees who worked at the 52 IPAW sites, 2 068 men and women completed the baseline questionnaire. IPAW was approved by and registered with the Danish Data Protection Agency (registration number: 2000-54-0066). Participants were provided information about the study and with the baseline questionnaire and responding was taken to imply informed consent to take part.

*Burnout, Motivation and Job Satisfaction study (Danish acronym: PUMA)*

Burnout, Motivation and Job Satisfaction study (Danish acronym: PUMA) is an intervention study of burn-out among employees in the human service sector (20). Selection criteria for the participating organisations was that they had between 200 and 500 employees, that occupational groups within each organisation were willing to participate and that the organisations would commit to the entire five-year study period. At study baseline in 1999-2000, 1 914 employees agreed to take part. Participants gave consent to having their national identity numbers collected and used in later record linkages to Danish hospitalisation and cause of death registries (Hospitalsindlæggelsesregisteret, Dødsårsagsregisteret. PUMA was approved by the Scientific Ethical Committees (Videnskabsetisk Komiteer) in the counties in which the study was conducted and approved by and registered with the Danish Data Protection Agency (registration number: 2000-54-0048).

*Still Working*

Still Working is an ongoing prospective cohort study. At study baseline in 1986, the employees (n = 12 173) at all Finnish centres of operation of Enso Gutzeit (a forestry products manufacturer) were invited to participate in a questionnaire survey on demographic, psychosocial and health-related factors and 9 282 individuals participated. The study was approved by the ethics committee of the Finnish Institute of Occupational Health (21, 22). Responding to the questionnaire voluntarily was taken to imply written consent.

*Whitehall II*

The Whitehall II study is a prospective cohort study set up to investigate socioeconomic determinants of health. At study baseline in 1985-1988, 10 308 civil service employees (6 895 men and 3 413 women) aged 35-55 and working in 20 civil service departments in London were invited to participate in the study (23, 24). The Whitehall II study protocol was approved by the University College London Medical School committee on the ethics of human research. Written informed consent was obtained at each data collection wave.

*WOLF (Work, Lipids, and Fibrinogen) Norrland and Stockholm studies*

The WOLF (Work, Lipids, and Fibrinogen) Norrland is a prospective cohort of 4 718 participants aged 19-65 working in companies in Jämtland and Västernorrland counties (25). WOLF Stockholm study is a prospective cohort study of 5 698 people (3 239 men and 2 459 women) aged 19–70 and working in companies in Stockholm county (26). At study baseline the participants underwent a clinical examination and completed a set of health questionnaires. For WOLF Stockholm, the baseline assessment was undertaken at 20 occupational health units between November 1992 and June 1995 and for WOLF Norrland at 13 occupational health service units in 1996-98. The Regional Research Ethics Board in Stockholm, and the ethics committee at Karolinska Institutet, Stockholm, Sweden approved the study. The participants received written and verbal information about the study and participation was voluntary. Answering the baseline questionnaire was taken to imply informed consent to participate.

**References**

1. Fransson EI, Heikkilä K, Nyberg ST, Zins M, Westerlund H, Westerholm P, et al. Job strain as a Risk Factor for Sedentary Lifestyle: An Individual-Participant Meta-analysis of up to 170 000 Men and Women. The IPD-Work Consortium. *American Journal of Epidemiology* 2012;**In press**.

2. Fransson EI, Nyberg ST, Heikkila K, Alfredsson L, Bacquer de D, Batty GD, et al. Comparison of alternative versions of the job demand-control scales in 17 European cohort studies: the IPD-Work consortium. *BMC Public Health* 2012;**12**(62).

3. Heikkila K, Nyberg ST, Fransson EI, Alfredsson L, De Bacquer D, Bjorner JB, et al. Job Strain and Alcohol Intake: A Collaborative Meta-analysis of Individual-participant Data from 140 000 Men and Women. *PLoS ONE* 2012;**7**(7):e40101.

4. Heikkilä K, Nyberg ST, Fransson EI, Alfredsson L, De Bacquer D, Bjorner JB, et al. Job Strain and Tobacco Smoking: An Individual-participant Data Meta-analysis of 166 130 Adults in 15 European Studies. *PLoS ONE* 2012;**7**(7):e35463.

5. Nyberg ST, Heikkila K, Fransson EI, Alfredsson L, De Bacquer D, Bjorner JB, et al. Job strain in relation to body mass index: pooled analysis of 160 000 adults from 13 cohort studies. *Journal of Internal Medicine* 2012;**272**(1):65-73.

6. Nyberg ST, Fransson EI, Heikkila K, Alfredsson L, Casini A, Clays E, et al. Job strain and cardiovascular disease risk factors: meta-analysis of individual-participant data from 47,000 men and women. *PLoS ONE* 2013;**8**(6):e67323.

7. Siegrist J, Dragano N, Nyberg ST, Lunau T, Alfredsson L, Erbel R, et al. Validating abbreviated measures of effort-reward imbalance at work in European cohort studies: the IPD-Work consortium. *Int Arch Occup Environ Health* 2013.

8. Heikkila K, Nyberg ST, Theorell T, Fransson EI, Alfredsson L, Bjorner JB, et al. Work Stress and Cancer Risk: A Meta-analysis of 5 700 Incident Cancer Events in 116 000 European Men and Women. *British Medical Journal* 2013;**346**:f165.

9. Kivimaki M, Nyberg ST, Batty GD, Fransson E, Heikkila K, Alfredsson L, et al. Job strain as a risk factor for future coronary heart disease: Collaborative meta-analysis of 2358 events in 197,473 men and women. *Lancet* 2012;**380**(9852):1491-1497.

10. Virtanen M, Heikkila K, Jokela M, Ferrie JE, Batty GD, Vahtera J, et al. Long working hours and coronary heart disease: a systematic review and meta-analysis. *Am J Epidemiol* 2012;**176**(7):586-596.

11. Kristensen TS, Hannerz H, Hogh A, Borg V. The Copenhagen Psychosocial Questionnaire--a tool for the assessment and improvement of the psychosocial work environment. *Scand J Work Environ Health* 2005;**31**(6):438-449.

12. Pejtersen JH, Kristensen TSnr, Borg V, Bjorner JB. The second version of the Copenhagen Psychosocial Questionnaire. *Scandinavian Journal of Public Health* 2010;**38**(3 suppl):8-24.

13. Burr H, Bjorner JB, Kristensen TS, Tüchsen F, Bach E. Trends in the Danish work environment in 1990–2000 and their associations with labor-force changes. *Scand J Work Environ Health* 2003;**29**(4):270-279.

14. Feveile H, Olsen O, Burr H, Bach E. Danish Work Environment Cohort Study 2005: From idea to sampling design. *Statistics in Transition* 2007;**8**(3):441-458.

15. Kivimaki M, Lawlor DA, Smith GD, Kouvonen A, Virtanen M, Elovainio M, et al. Socioeconomic Position, Co-Occurrence of Behavior-Related Risk Factors, and Coronary Heart Disease: the Finnish Public Sector Study. *Am J Public Health* 2007;**97**(5):874-879.

16. Justice FMo. Personal Data Act (523/1999). In; 1999.

17. Korkeila K, Suominen S, Ahvenainen J, Ojanlatva A, Rautava P, Helenius H, et al. Non-response and related factors in a nation-wide health survey. *Eur J Epidemiol* 2001;**17**(11):991-999.

18. Nielsen M, Kristensen T, Smith-Hansen L. The Intervention Project on Absence and Well-being (IPAW): design and results from the baseline of a 5-year study. *Work and Stress* 2002;**16**:191-206.

19. Nielsen ML, Rugulies R, Christensen KB, Smith-Hansen L, Bjorner JB, Kristensen T. Impact of the psychosocial work environment on registered absence from work: a two-year longitudinal study using the IPAW cohort. *Work & Stress* 2004;**18**(4):323-335.

20. Borritz M, Rugulies R, Bjorner JB, Villadsen E, Mikkelsen OA, Kristensen TS. Burnout among employees in human service work: design and baseline findings of the PUMA study. *Scand J Public Health* 2006;**34**(1):49-58.

21. Kalimo R, Toppinen S. Organizational well-being: ten years of research and development: in a forest industry corporation. In: Kompier M, Cooper C, editors. Preventing Stress, Improving Productivity: European Case Studies in the Workplace. London: Routledge; 1999. p. 52-85.

22. Vaananen A, Murray M, Koskinen A, Vahtera J, Kouvonen A, Kivimaki M. Engagement in cultural activities and cause-specific mortality: prospective cohort study. *Prev Med* 2009;**49**(2-3):142-147.

23. Marmot M, Brunner E. Cohort Profile: the Whitehall II study. *Int J Epidemiol* 2005;**34**(2):251-256.

24. Marmot MG, Smith GD, Stansfeld S, Patel C, North F, Head J, et al. Health inequalities among British civil servants: the Whitehall II study. *Lancet* 1991;**337**(8754):1387-1393.

25. Alfredsson L, Hammar N, Fransson E, de Faire U, Hallqvist J, Knutsson A, et al. Job strain and major risk factors for coronary heart disease among employed males and females in a Swedish study on work, lipids and fibrinogen. *Scand J Work Environ Health* 2002;**28**(4):238-248.

26. Peter R, Alfredsson L, Hammar N, Siegrist J, Theorell T, P. W. High effort, low reward, and cardiovascular risk factors in employed Swedish men and women: baseline results from the WOLF Study. *J Epidemiol Community Health* 1998;**52**:540-547
