# Supplementary material for: Job strain and the risk of severe asthma exacerbations: a meta‐analysis of individual‐participant data from 100 000 European men and women
Source: Allergy. 2014 Apr 12;69(6):775–83. doi: 10.1111/all.12381 (PMC4114530; doi:10.1111/all.12381)
Supplement: Supplementary file 2 — Appendix S2. Potential confounders. [file all-69-775-s5.doc]

**Appendix 2. Potential confounders**

We adjusted our analyses for age, sex, socioeconomic position, body mass index (BMI), tobacco smoking and alcohol intake. Information on sex and age was obtained from population registries or interview (COPSOQ-I, COPSOQ-II, DWECS, FPS, IPAW, PUMA, Still Working, WOLF Norrland and WOLF Stockholm) or from participant-completed questionnaires (in HeSSup and Whitehall II). Socioeconomic position was based on occupation, which was ascertained from the employers' or other registers (in COPSOQ-I, COPSOQ-II, DWECS, FPS, IPAW, PUMA and Still Working) or participant-completed questionnaires (in HeSSup Whitehall II, WOLF Norrland and WOLF Stockholm). In HeSSup, socioeconomic position was based on the highest educational qualification reported by each participant. We harmonised socioeconomic position into low (routine and manual occupations or comprehensive education), intermediate (non-manual intermediate occupations or vocational education), high (higher managerial, administrative and professional occupations or university-level education) and other (for those with missing data on job title) (1).

Smoking and alcohol intake were participant-reported in all studies. Smoking was harmonised into never, ex- and current (2). Alcohol intake was ascertained from questions on the total number of alcoholic drinks (defined as one unit, one glass or 10g of ethanol) the participants consumed in a week. Alcohol intake was harmonised into none, moderate (women: 1-14 drinks/week, men: 1-21 drinks/week), intermediate (women: 15-20 drinks/week, men: 22-27 drinks/week) and heavy (women: >=21 drinks/wk, men: >=28 drinks/week) (3). In Still Working, we were only able to categorise alcohol intake approximately as none, moderate and heavy. Body mass index (BMI: weight in kilograms divided by height in meters squared) was calculated using data on height and weight, which were self-reported in six studies (COPSOQ-II, DWECS, FPS, HeSSup, IPAW and PUMA) and measured in three studies (Whitehall II, WOLF Norrland and WOLF Stockholm). We harmonised BMI according to the World Health Organization recommendations into underweight (<18.5 kg/m^2^), normal weight (18.5-24.9), overweight (25-29.9) and obese (>=30)(4). Participants with BMI <15 or >50 were excluded from the analysis. In COPSOQ-I no data were collected on BMI and alcohol intake. Similarly, no BMI data were collected in Still Working. The multivariable-adjusted analyses in these studies were adjusted for all other covariates apart from the ones that were not available.

**References**

1. Nyberg ST, Heikkila K, Fransson EI, Alfredsson L, De Bacquer D, Bjorner JB, et al. Job strain in relation to body mass index: pooled analysis of 160 000 adults from 13 cohort studies. *Journal of Internal Medicine* 2012;**272**(1):65-73.

2. Heikkilä K, Nyberg ST, Fransson EI, Alfredsson L, De Bacquer D, Bjorner JB, et al. Job Strain and Tobacco Smoking: An Individual-participant Data Meta-analysis of 166 130 Adults in 15 European Studies. *PLoS ONE* 2012;**7**(7):e35463.

3. Heikkila K, Nyberg ST, Fransson EI, Alfredsson L, De Bacquer D, Bjorner JB, et al. Job Strain and Alcohol Intake: A Collaborative Meta-analysis of Individual-participant Data from 140 000 Men and Women. *PLoS ONE* 2012;**7**(7):e40101.

4. Obesity: preventing and managing the global epidemic. Report of a WHO consultation. *World Health Organ Tech Rep Ser* 2000;**894**:i-xii, 1-253.
