# Supplementary material for: Job strain and the risk of severe asthma exacerbations: a meta‐analysis of individual‐participant data from 100 000 European men and women
Source: Allergy. 2014 Apr 12;69(6):775–83. doi: 10.1111/all.12381 (PMC4114530; doi:10.1111/all.12381)
Supplement: Supplementary file 3 — Appendix S3. Additional results. [file all-69-775-s6.docx]

**Appendix 3. Additional results**

Table S1. Participant characteristics

| **Study, country** | **Baseline year** | **N with complete data^1^** | | **N (%) women** | | **Mean (SD) age at baseline** | | **N (%) baseline smokers** | |
| --- | --- | --- | --- | --- | --- | --- | --- | --- | --- |
|  |  | **Asthma as primary diagnostic code** | **Asthma as any diagnostic code** | **Asthma as primary diagnostic code** | **Asthma as any diagnostic code** | **Asthma as primary diagnostic code** | **Asthma as any diagnostic code** | **Asthma as primary diagnostic code** | **Asthma as any diagnostic code** |
| Copenhagen Psychosocial Questionnaire I (COPSOQ-I), Denmark | 1997 | 1 716 | 1 707 | 829 (48.3) | 824 (48.3) | 41.2 (10.6) | 41.2 (10.5) | 629 (36.7) | 626 (36.7) |
| Copenhagen Psychosocial Questionnaire II (COPSOQ-II), Denmark | 2004-2005 | 3 293 | 3 280 | 1 713 (52.0) | 1 707 (52.0) | 42.8 (10.2) | 42.8 (10.2) | 1 056 (32.1) | 1 048 (32.0) |
| Danish Work Environment Cohort Study (DWECS), Denmark | 2000 | 5 396 | 5 375 | 2 508 (46.5) | 2 501 (46.5) | 41.5 (10.9) | 41.6 (10.9) | 2 005 (37.2) | 1 997 (37.2) |
| Finnish Public Sector (FPS), Finland | 2000 | 44 007 | 43 652 | 35 473 (80.6) | 35 193 (80.6) | 44.6 (9.5) | 44.5 (9.4) | 7 867 (17.9) | 7 813 (17.9) |
| Health and Social Support (HeSSup), Finland | 1998 | 15 004 | 15 004 | 5 464 (56.4) | 8 464 (56.4) | 39.6 (10.3) | 39.6 (10.3) | 3 934 (26.2) | 3 934 (26.2) |
| Intervention Project on Absence and Well-being (IPAW), Denmark | 1996-1997 | 1 912 | 1 905 | 1 267 (66.3) | 1 262 (66.3) | 40.7 (10.5) | 40.7 (10.5) | 902 (47.2) | 899 (47.2) |
| Burnout, Motivation and Job Satisfaction study (Danish acronym PUMA), Denmark | 1999-2000 | 1 723 | 1 716 | 1 413 (82.0) | 1 407 (82.0) | 42.7 (10.6) | 42.7 (10.2) | 677 (39.3) | 676 (39.4) |
| Still Working, Finland | 1986 | 8 916 | 8 911 | 2 030 (22.8) | 2 028 (22.8) | 40.9 (9.1) | 40.9 (9.1) | 2 951 (33.1) | 2 949 (33.1) |
| Whitehall II, United Kingdom | 1985–1988 | 10 175 | 10 175 | 3 368 (33.1) | 3 368 (33.1) | 44.4 (6.0) | 44.4 (6.0) | 1 876 (18.4) | 1 876 (18.4) |
| Work Lipids and Fibrinogen (WOLF) Norrland, Sweden | 1996–1998 | 4 561 | 4 556 | 750 (16.4) | 749 (16.4) | 44.0 (10.2) | 44.0 (10.3) | 860 (18.9) | 860 (18.9) |
| Work Lipids and Fibrinogen (WOLF) Stockholm, Sweden | 1992–1995 | 5 472 | 5 464 | 2 359 (43.1) | 2 358 (43.2) | 41.5 (11.0) | 41.5 (11.0) | 1 374 (25.1) | 1 372 (25.1) |
| **All** | **1985 - 2005** | **102 175** | **101 745** | **57 147 (55.9)** | **59 861 (56.2)** | **42.9** | **42.9** | **23 831 (23.3)** | **24 050 (23.6)** |

^1^ Participants with complete data on job strain, asthma and covariates.

SD: standard deviation

Figure S1. Age and sex-adjusted associations between job strain model quadrants and asthma exacerbations (hospitalisation or death), with asthma as primary diagnostic code

NOTE: Weights are from random effects analysis

.

.

.

**Passive job**

COPSOQ-I

COPSOQ-II

DWECS

FPS

HeSSup

IPAW

PUMA

Still Working

WOLF Norrland

WOLF Stockholm

Whitehall II

Random effects estimate (I^2^ = 0.0%, p = 0.8)

Fixed effect estimate

**Active job**

COPSOQ-I

COPSOQ-II

DWECS

FPS

HeSSup

IPAW

PUMA

Still Working

WOLF Stockholm

Whitehall II

WOLF Norrland

Random effects estimate (I^2^ = 0.0%, p = 0.6)

Fixed effect estimate

**High strain**

COPSOQ-I

COPSOQ-II

DWECS

FPS

HeSSup

IPAW

PUMA

Still Working

WOLF Norrland

WOLF Stockholm

Whitehall II

Random effects estimate (I^2^ = 0.0%, p = 0.7)

Fixed effect estimate

Study

439

965

1037

12395

4030

392

588

2713

1114

1786

3352

384

669

1388

9995

3911

606

298

1814

1456

2982

1425

348

463

1196

7052

2644

330

257

1390

581

873

1421

Participants

5

1

8

33

11

5

7

21

3

3

20

8

5

9

37

13

11

5

12

3

6

0

3

3

8

32

9

7

4

10

2

1

9

Asthma exacerbations

1.02 (0.31, 3.35)

0.21 (0.02, 1.71)

1.01 (0.42, 2.44)

1.27 (0.78, 2.09)

0.94 (0.42, 2.11)

0.52 (0.19, 1.43)

1.15 (0.39, 3.45)

1.42 (0.72, 2.78)

1.31 (0.26, 6.51)

0.99 (0.16, 6.08)

1.67 (0.69, 4.05)

1.12 (0.86, 1.47)

1.12 (0.86, 1.47)

1.94 (0.67, 5.61)

1.45 (0.44, 4.79)

0.87 (0.37, 2.04)

1.80 (1.11, 2.92)

1.09 (0.51, 2.36)

0.70 (0.32, 1.52)

1.59 (0.48, 5.23)

1.29 (0.60, 2.76)

1.43 (0.24, 8.64)

0.71 (0.24, 2.11)

(Excluded)

1.27 (0.97, 1.65)

1.27 (0.97, 1.65)

0.75 (0.19, 3.01)

1.25 (0.31, 5.03)

0.85 (0.35, 2.05)

2.11 (1.28, 3.49)

1.05 (0.45, 2.47)

0.80 (0.33, 1.98)

1.47 (0.41, 5.23)

1.29 (0.58, 2.90)

1.66 (0.28, 9.99)

0.70 (0.06, 7.80)

2.00 (0.74, 5.43)

1.37 (1.03, 1.82)

1.37 (1.03, 1.82)

HR (95% CI)

5.11

1.61

9.34

29.53

11.12

7.04

6.04

15.93

2.82

2.21

9.24

100.00

6.28

4.96

9.70

30.29

11.90

11.58

4.98

12.21

2.18

5.92

0.00

100.00

4.14

4.11

10.14

31.80

10.92

9.87

4.96

12.22

2.47

1.37

8.00

100.00

% Weight

1.02 (0.31, 3.35)

0.21 (0.02, 1.71)

1.01 (0.42, 2.44)

1.27 (0.78, 2.09)

0.94 (0.42, 2.11)

0.52 (0.19, 1.43)

1.15 (0.39, 3.45)

1.42 (0.72, 2.78)

1.31 (0.26, 6.51)

0.99 (0.16, 6.08)

1.67 (0.69, 4.05)

1.12 (0.86, 1.47)

1.12 (0.86, 1.47)

1.94 (0.67, 5.61)

1.45 (0.44, 4.79)

0.87 (0.37, 2.04)

1.80 (1.11, 2.92)

1.09 (0.51, 2.36)

0.70 (0.32, 1.52)

1.59 (0.48, 5.23)

1.29 (0.60, 2.76)

1.43 (0.24, 8.64)

0.71 (0.24, 2.11)

(Excluded)

1.27 (0.97, 1.65)

1.27 (0.97, 1.65)

0.75 (0.19, 3.01)

1.25 (0.31, 5.03)

0.85 (0.35, 2.05)

2.11 (1.28, 3.49)

1.05 (0.45, 2.47)

0.80 (0.33, 1.98)

1.47 (0.41, 5.23)

1.29 (0.58, 2.90)

1.66 (0.28, 9.99)

0.70 (0.06, 7.80)

2.00 (0.74, 5.43)

1.37 (1.03, 1.82)

1.37 (1.03, 1.82)

5.11

1.61

9.34

29.53

11.12

7.04

6.04

15.93

2.82

2.21

9.24

100.00

6.28

4.96

9.70

30.29

11.90

11.58

4.98

12.21

2.18

5.92

0.00

100.00

4.14

4.11

10.14

31.80

10.92

9.87

4.96

12.22

2.47

1.37

8.00

100.00

1

.25

.5

1

2.5

5

HR: hazard ratio, CI: confidence intervalFigure S2. Age and sex-adjusted associations between job strain model quadrants and asthma exacerbations (hospitalisation or death), with asthma as any diagnostic code

NOTE: Weights are from random effects analysis

.

.

.

Passive job

COPSOQ-I

COPSOQ-II

DWECS

FPS

HeSSup

IPAW

PUMA

Still Working

WOLF Norrland

WOLF Stockholm

Whitehall II

Random effects estimate (I^2^ = 0.0%, p = 0.8)

Fixed effect estimate

Active job

COPSOQ-I

COPSOQ-II

DWECS

FPS

HeSSup

IPAW

PUMA

Still Working

WOLF Norrland

WOLF Stockholm

Whitehall II

Random effects estimate (I^2^ = 0.0%, p = 0.8)

Fixed effect estimate

High strain

COPSOQ-I

COPSOQ-II

DWECS

FPS

HeSSup

IPAW

PUMA

Still Working

WOLF Norrland

WOLF Stockholm

Whitehall II

Random effects estimate (I^2^ = 0.0%, p = 0.9)

Fixed effect estimate

Study

433

964

1035

12287

4030

392

586

2711

1114

1785

3352

383

665

1383

9910

3911

603

295

1814

1424

1453

2982

348

461

1194

6993

2644

330

256

1387

579

871

1421

Participants

5

1

8

100

11

5

6

42

7

15

154

8

4

9

97

13

10

4

21

4

14

102

3

2

8

67

9

7

4

21

3

7

54

Asthma exacerbations

1.03 (0.31, 3.39)

0.24 (0.03, 2.05)

1.19 (0.48, 2.96)

1.08 (0.82, 1.42)

0.88 (0.40, 1.95)

0.51 (0.19, 1.42)

1.01 (0.32, 3.14)

1.31 (0.82, 2.09)

1.39 (0.49, 3.97)

1.45 (0.61, 3.42)

1.23 (0.92, 1.64)

1.13 (0.96, 1.33)

1.13 (0.96, 1.33)

1.94 (0.67, 5.60)

1.37 (0.37, 5.13)

1.04 (0.43, 2.52)

1.32 (1.00, 1.74)

1.01 (0.48, 2.16)

0.63 (0.28, 1.41)

1.28 (0.36, 4.54)

1.04 (0.60, 1.81)

0.61 (0.18, 2.08)

1.61 (0.67, 3.84)

1.16 (0.86, 1.56)

1.18 (1.00, 1.39)

1.18 (1.00, 1.39)

0.75 (0.19, 2.99)

0.98 (0.19, 5.07)

1.02 (0.41, 2.55)

1.25 (0.92, 1.70)

0.98 (0.42, 2.28)

0.80 (0.33, 1.97)

1.50 (0.42, 5.31)

1.28 (0.73, 2.22)

1.23 (0.32, 4.80)

1.35 (0.49, 3.73)

1.17 (0.82, 1.67)

1.17 (0.97, 1.42)

1.17 (0.97, 1.42)

HR (95% CI)

1.86

0.57

3.19

35.50

4.19

2.57

2.05

12.15

2.40

3.57

31.95

100.00

2.46

1.58

3.54

36.43

4.83

4.29

1.72

9.20

1.82

3.64

30.49

100.00

1.81

1.29

4.16

37.03

4.93

4.32

2.17

11.40

1.89

3.37

27.64

100.00

% Weight

1.03 (0.31, 3.39)

0.24 (0.03, 2.05)

1.19 (0.48, 2.96)

1.08 (0.82, 1.42)

0.88 (0.40, 1.95)

0.51 (0.19, 1.42)

1.01 (0.32, 3.14)

1.31 (0.82, 2.09)

1.39 (0.49, 3.97)

1.45 (0.61, 3.42)

1.23 (0.92, 1.64)

1.13 (0.96, 1.33)

1.13 (0.96, 1.33)

1.94 (0.67, 5.60)

1.37 (0.37, 5.13)

1.04 (0.43, 2.52)

1.32 (1.00, 1.74)

1.01 (0.48, 2.16)

0.63 (0.28, 1.41)

1.28 (0.36, 4.54)

1.04 (0.60, 1.81)

0.61 (0.18, 2.08)

1.61 (0.67, 3.84)

1.16 (0.86, 1.56)

1.18 (1.00, 1.39)

1.18 (1.00, 1.39)

0.75 (0.19, 2.99)

0.98 (0.19, 5.07)

1.02 (0.41, 2.55)

1.25 (0.92, 1.70)

0.98 (0.42, 2.28)

0.80 (0.33, 1.97)

1.50 (0.42, 5.31)

1.28 (0.73, 2.22)

1.23 (0.32, 4.80)

1.35 (0.49, 3.73)

1.17 (0.82, 1.67)

1.17 (0.97, 1.42)

1.17 (0.97, 1.42)

1.86

0.57

3.19

35.50

4.19

2.57

2.05

12.15

2.40

3.57

31.95

100.00

2.46

1.58

3.54

36.43

4.83

4.29

1.72

9.20

1.82

3.64

30.49

100.00

1.81

1.29

4.16

37.03

4.93

4.32

2.17

11.40

1.89

3.37

27.64

100.00

1

.25

.5

1

2.5

5

HR: hazard ratio, CI: confidence interval Figure S3. Multivariable-adjusted* associations between job strain model quadrants and asthma exacerbations (hospitalisation or death), with asthma as primary diagnostic code

NOTE: Weights are from random effects analysis

.

.

.

Passive job

COPSOQ-I

COPSOQ-II

DWECS

FPS

HeSSup

IPAW

PUMA

Still Working

WOLF Norrland

WOLF Stockholm

Whitehall II

Random effects estimate (I^2^ = 0.0%, p = 0.8)

Fixed effect estimate

Active job

COPSOQ-I

COPSOQ-II

DWECS

FPS

HeSSup

IPAW

PUMA

Still Working

WOLF Stockholm

Whitehall II

WOLF Norrland

Random effects estimate (I^2^ = 0.0%, p = 0.6)

Fixed effect estimate

High strain

COPSOQ-I

COPSOQ-II

DWECS

FPS

HeSSup

IPAW

PUMA

Still Working

WOLF Norrland

WOLF Stockholm

Whitehall II

Random effects (I^2^ = 0.0%, p = 0.6)

Fixed effect estimate

Study

439

965

1037

12395

4030

392

588

2713

1114

1786

3352

384

669

1388

9995

3911

606

298

1814

1456

2982

1425

348

463

1196

7052

2644

330

257

1390

581

873

1421

Participants

5

1

8

33

11

5

7

21

3

3

20

8

5

9

37

13

11

5

12

3

6

0

3

3

8

32

9

7

4

10

2

1

9

Asthma exacerbations

0.96 (0.29, 3.24)

0.18 (0.02, 1.55)

0.97 (0.40, 2.38)

1.26 (0.75, 2.11)

0.96 (0.42, 2.18)

0.51 (0.18, 1.41)

0.96 (0.32, 2.90)

1.19 (0.60, 2.36)

1.25 (0.25, 6.39)

0.79 (0.12, 5.22)

1.41 (0.56, 3.59)

1.04 (0.79, 1.37)

1.04 (0.79, 1.37)

1.76 (0.61, 5.12)

1.42 (0.43, 4.75)

0.87 (0.37, 2.05)

1.79 (1.10, 2.91)

1.04 (0.48, 2.26)

0.67 (0.31, 1.46)

1.94 (0.58, 6.46)

1.45 (0.68, 3.12)

1.40 (0.23, 8.68)

0.76 (0.25, 2.31)

(Excluded)

1.28 (0.98, 1.67)

1.28 (0.98, 1.67)

0.69 (0.17, 2.81)

1.37 (0.33, 5.71)

0.77 (0.31, 1.92)

2.11 (1.26, 3.55)

1.00 (0.42, 2.41)

0.79 (0.32, 1.98)

1.08 (0.30, 3.93)

1.12 (0.50, 2.53)

1.67 (0.27, 10.19)

0.57 (0.05, 6.61)

1.76 (0.64, 4.84)

1.28 (0.96, 1.71)

1.28 (0.96, 1.71)

HR (95% CI)

5.20

1.67

9.53

28.69

11.30

7.28

6.24

16.25

2.89

2.14

8.82

100.00

6.30

4.92

9.76

30.40

11.91

11.61

4.92

12.21

2.14

5.82

0.00

100.00

4.22

4.08

10.07

30.90

10.91

10.05

5.01

12.61

2.55

1.40

8.18

100.00

% Weight

0.96 (0.29, 3.24)

0.18 (0.02, 1.55)

0.97 (0.40, 2.38)

1.26 (0.75, 2.11)

0.96 (0.42, 2.18)

0.51 (0.18, 1.41)

0.96 (0.32, 2.90)

1.19 (0.60, 2.36)

1.25 (0.25, 6.39)

0.79 (0.12, 5.22)

1.41 (0.56, 3.59)

1.04 (0.79, 1.37)

1.04 (0.79, 1.37)

1.76 (0.61, 5.12)

1.42 (0.43, 4.75)

0.87 (0.37, 2.05)

1.79 (1.10, 2.91)

1.04 (0.48, 2.26)

0.67 (0.31, 1.46)

1.94 (0.58, 6.46)

1.45 (0.68, 3.12)

1.40 (0.23, 8.68)

0.76 (0.25, 2.31)

(Excluded)

1.28 (0.98, 1.67)

1.28 (0.98, 1.67)

0.69 (0.17, 2.81)

1.37 (0.33, 5.71)

0.77 (0.31, 1.92)

2.11 (1.26, 3.55)

1.00 (0.42, 2.41)

0.79 (0.32, 1.98)

1.08 (0.30, 3.93)

1.12 (0.50, 2.53)

1.67 (0.27, 10.19)

0.57 (0.05, 6.61)

1.76 (0.64, 4.84)

1.28 (0.96, 1.71)

1.28 (0.96, 1.71)

5.20

1.67

9.53

28.69

11.30

7.28

6.24

16.25

2.89

2.14

8.82

100.00

6.30

4.92

9.76

30.40

11.91

11.61

4.92

12.21

2.14

5.82

0.00

100.00

4.22

4.08

10.07

30.90

10.91

10.05

5.01

12.61

2.55

1.40

8.18

100.00

1

.25

.5

1

3

6

* Adjusted for age, sex, socioeconomic position, body mass index, smoking and alcohol intake.

HR: hazard ratio, CI: confidence interval Figure S4. Multivariable-adjusted* associations between job strain model quadrants and asthma exacerbations (hospitalisation or death), with asthma as any diagnostic code

NOTE: Weights are from random effects analysis

.

.

.

**Passive job**

COPSOQ-I

COPSOQ-II

DWECS

FPS

HeSSup

IPAW

PUMA

Still Working

WOLF Norrland

WOLF Stockholm

Whitehall II

Random effects estimate (I^2^ = 0.0%, p = 0.9)

Fixed effect estimate

**Active job**

COPSOQ-I

COPSOQ-II

DWECS

FPS

HeSSup

IPAW

PUMA

Still Working

WOLF Norrland

WOLF Stockholm

Whitehall II

Random effects estimate (I^2^ = 0.0%, p = 0.8)

Fixed effect estimate

**High strain**

COPSOQ-I

COPSOQ-II

DWECS

FPS

HeSSup

IPAW

PUMA

Still Working

WOLF Norrland

WOLF Stockholm

Whitehall II

Random effects estimate (I^2^ = 0.0%, p = 0.9)

Fixed effect estimate

Study

433

964

1035

12287

4030

392

586

2711

1114

1785

3352

383

665

1383

9910

3911

603

295

1814

1424

1453

2982

348

461

1194

6993

2644

330

256

1387

579

871

1421

Participants

5

1

8

100

11

5

6

42

7

15

154

8

4

9

97

13

10

4

21

4

14

102

3

2

8

67

9

7

4

21

3

7

54

Asthma exacerbations

0.97 (0.29, 3.27)

0.20 (0.02, 1.81)

1.16 (0.46, 2.93)

0.99 (0.74, 1.32)

0.88 (0.39, 1.99)

0.51 (0.18, 1.43)

0.86 (0.27, 2.73)

1.09 (0.68, 1.75)

1.62 (0.55, 4.77)

1.04 (0.42, 2.57)

1.01 (0.75, 1.38)

0.99 (0.84, 1.18)

0.99 (0.84, 1.18)

1.76 (0.61, 5.12)

1.47 (0.39, 5.65)

1.04 (0.43, 2.51)

1.35 (1.03, 1.78)

0.96 (0.45, 2.06)

0.61 (0.27, 1.36)

1.53 (0.43, 5.52)

1.17 (0.67, 2.03)

0.66 (0.19, 2.37)

1.68 (0.70, 4.05)

1.28 (0.94, 1.74)

1.24 (1.05, 1.47)

1.24 (1.05, 1.47)

0.69 (0.17, 2.80)

1.01 (0.19, 5.47)

0.94 (0.37, 2.42)

1.16 (0.85, 1.60)

0.93 (0.39, 2.20)

0.80 (0.32, 2.00)

1.12 (0.31, 4.10)

1.10 (0.63, 1.93)

1.20 (0.30, 4.75)

1.11 (0.40, 3.12)

1.03 (0.72, 1.48)

1.07 (0.88, 1.29)

1.07 (0.88, 1.29)

HR (95% CI)

1.95

0.60

3.33

35.33

4.37

2.72

2.17

12.70

2.46

3.55

30.80

100.00

2.48

1.56

3.58

36.86

4.87

4.33

1.72

9.32

1.74

3.63

29.91

100.00

1.84

1.28

4.10

36.22

4.89

4.37

2.16

11.71

1.91

3.43

28.09

100.00

% Weight

0.97 (0.29, 3.27)

0.20 (0.02, 1.81)

1.16 (0.46, 2.93)

0.99 (0.74, 1.32)

0.88 (0.39, 1.99)

0.51 (0.18, 1.43)

0.86 (0.27, 2.73)

1.09 (0.68, 1.75)

1.62 (0.55, 4.77)

1.04 (0.42, 2.57)

1.01 (0.75, 1.38)

0.99 (0.84, 1.18)

0.99 (0.84, 1.18)

1.76 (0.61, 5.12)

1.47 (0.39, 5.65)

1.04 (0.43, 2.51)

1.35 (1.03, 1.78)

0.96 (0.45, 2.06)

0.61 (0.27, 1.36)

1.53 (0.43, 5.52)

1.17 (0.67, 2.03)

0.66 (0.19, 2.37)

1.68 (0.70, 4.05)

1.28 (0.94, 1.74)

1.24 (1.05, 1.47)

1.24 (1.05, 1.47)

0.69 (0.17, 2.80)

1.01 (0.19, 5.47)

0.94 (0.37, 2.42)

1.16 (0.85, 1.60)

0.93 (0.39, 2.20)

0.80 (0.32, 2.00)

1.12 (0.31, 4.10)

1.10 (0.63, 1.93)

1.20 (0.30, 4.75)

1.11 (0.40, 3.12)

1.03 (0.72, 1.48)

1.07 (0.88, 1.29)

1.07 (0.88, 1.29)

1.95

0.60

3.33

35.33

4.37

2.72

2.17

12.70

2.46

3.55

30.80

100.00

2.48

1.56

3.58

36.86

4.87

4.33

1.72

9.32

1.74

3.63

29.91

100.00

1.84

1.28

4.10

36.22

4.89

4.37

2.16

11.71

1.91

3.43

28.09

100.00

1

.25

.5

1

3

6

* Adjusted for age, sex, socioeconomic position, body mass index, smoking and alcohol intake.

HR: hazard ratio, CI: confidence interval

Table S2. Associations between job strain and asthma exacerbations (hospitalisation or death), stratified by baseline smoking

| **Exposure** | **Stratum** | **Random effects HR (95% CI) for asthma exacerbation** | | | |
| --- | --- | --- | --- | --- | --- |
|  |  | **Age and sex-adjusted** | | **Multivariable adjusted** | |
|  |  | **Asthma as primary diagnostic code** | **Asthma as any diagnostic code** | **Asthma as primary diagnostic code** | **Asthma as any diagnostic code** |
| Job strain | Baseline smokers | 0.85 (0.38, 1.92) | 0.88 (0.62, 1.24) | 0.83 (0.37, 1.87) | 0.83 (0.58, 1.17) |
|  | Baseline non-smokers | 1.45 (1.01, 1.91) | 1.17 (0.99, 1.43) | 1.40 (1.05, 1.85) | 1.14 (0.95, 1.37) |

^1^ Adjusted for age, sex, socioeconomic position, body mass index and alcohol intake.

NB: some studies excluded from these analyses due to insufficient numbers of asthma exacerbations.

**Sensitivity analyses**

*Job strain quadrants and asthma exacerbations, stratified by baseline smoking*

We also ran the models of the association between active job and asthma exacerbations, stratified by baseline smoking. The associations between active job and asthma exacerbation as any diagnostic code were similar to our main findings in the analyses restricted to baseline non-smokers: the age and sex-adjusted (random effects HR was 1.22 (95% CI: 1.01, 1.48) and the multivariable-adjusted HR was 1.29 (95% CI: 1.06, 1.56). Due to the small number of baseline smokers who experienced severe asthma exacerbations during the follow-up, the stratified analyses in this group were considerably underpowered and the findings therefore difficult to interpret. Having an active job was not associated with severe asthma exacerbations in baseline smokers in the age and sex adjusted analyses (random effects HR: 19.45, 95% CI: 0.42, 911.79) or multivariable-adjusted analyses (random effects HR: 18.83, 95% CI: 0.45, 779.80). The findings of the analyses of the other job strain quadrants were similar to those of our main analyses but again, due to the small number of baseline smokers who had asthma exacerbations during follow-up, the stratified analyses in this group were underpowered.

*Heterogeneity*

There was little heterogeneity among the study-specific association estimates in any of our meta-analyses. We used meta-regression to explore whether the association estimates differed in the two studies with long follow-up (median>20 years: Still Working and Whitehall II) and studies with short follow-up (the rest of the studies). There was no evidence that the associations between job strain and severe asthma exacerbations were different: age and sex-adjusted ratio of hazard ratios (RHR) comparing long to shorter (median < 20 years) follow-up was 0.97 (95% confidence interval: 0.72, 1.32) and the corresponding multivariable-adjusted RHR was 0.95 (95% confidence interval: 0.70, 1.29).

*Hospital episodes data in Whitehall II*

As the hospital episodes data we used to define severe asthma exacerbations may not have been complete in the United Kingdom until 2003, we conducted sensitivity analyses in our United Kingdom study, Whitehall II. We moved the analytical baseline from the study baseline (phase 1 of the study) to 2003-04 (phase 7) and ran the same age and sex-adjusted and multivariable adjusted models with the new baseline. However, although the study-specific association estimates were smaller and the analyses less well powered, the meta-analysis estimates from the sensitivity analyses were similar to our main findings (Table S3).

Table S3. Sensitivity analysis: comparing associations of job strain with severe asthma exacerbations (hospitalisation or death) in Whitehall II, with the follow-up beginning at the official study baseline and with the follow-up moved beginning in 2003-04

| **Beginning of follow-up** | **N with complete data** | **N asthma** | **Study specific estimates** | | **Random effects overall estimates, HR (95% CI)** | |
| --- | --- | --- | --- | --- | --- | --- |
|  |  |  | **Age and sex-adjusted HR (95% CI)** | **Multivariable-adjusted* HR (95% CI)** | **Age and sex-adjusted HR (95% CI)** | **Multivariable-adjusted* HR (95% CI)** |
| **Asthma as primary diagnostic code** | | | | | | |
| 1985–88 | 10 175 | 42 | 1.64 (0.78, 3.43) | 1.52 (0.72, 3.20) | 1.27 (1.00, 1.61) | 1.22 (0.96, 1.55) |
| 2003-04 | 3 025 | 15 | 0.81 (0.18, 3.61) | 0.85 (0.19, 3.85) | 1.22 (0.96, 1.56) | 1.18 (0.92, 1.52) |
| **Asthma as any diagnostic code** | | | | | | |
| 1985–88 | 10 175 | 382 | 1.01 (0.76, 1.35) | 0.97 (0.72, 1.29) | 1.06 (0.91, 1.25) | 1.01 (0.86, 1.19) |
| 2003-04 | 3 025 | 97 | 0.49 (0.24, 1.01) | 0.49 (0.23, 1.01) | 1.04 (0.86, 1.24) | 0.99 (0.82, 1.19) |

* Adjusted for age, sex, socioeconomic position, body mass index, smoking and alcohol intake.
